# True Left-Sided Gallbladder: A Case Report of Rare Incidental Findings During Cholecystectomy in a Jordanian Patient

**DOI:** 10.7759/cureus.75301

**Published:** 2024-12-07

**Authors:** Mo'taz F Naffa', Mahmoud A Al-Hurani, Yazan O Al-Khawaled, Yazan Y Albaddawi, Ahmad M Alfalahat

**Affiliations:** 1 General and Hepatopancreaticbiliary Surgery, Jordanian Royal Medical Services, Amman, JOR; 2 General Surgery, Jordanian Royal Medical Services, Amman, JOR

**Keywords:** biliary anomaly, gallbladder variations, laparoscopic cholecystectomy, safe laparoscopic cholecystectomy, true left-sided gallbladder

## Abstract

The biliary system exhibits significant anatomical variations, which pose challenges for most surgeons during cholecystectomy. Among these variations, a true left-sided gallbladder (LSG) is an uncommon finding. In such cases, the gallbladder is located to the left of the round ligament. Although it can be diagnosed by preoperative imaging, such as magnetic resonance imaging and computed tomography, true LSG is diagnosed intraoperatively in most reported cases.

A 33-year-old man with no medical or surgical history presented with recurrent attacks of typical biliary colic and was admitted for elective laparoscopic cholecystectomy after abdominal ultrasonography showed a single large gallbladder stone that measured 3.3 cm. No further imaging studies were performed because the patient’s presentation, physical examination, and laboratory results did not indicate any biliary tree obstruction or suspicious biliary anomaly. Intraoperatively, the gallbladder was not found in its normal anatomical position, and the diagnosis of true LSG was confirmed when the gallbladder was detected to the left of the round ligament. The surgery was completed safely using a standard laparoscopic approach. The patient was discharged home on the second postoperative day.

True LSG is the most common variant of LSG without situs viscerum inversus (woSVI). The presentation of true LSG is similar to that of a normally positioned right-sided gallbladder. In most cases, it is discovered during surgery and may necessitate modifications to the surgical approach, such as adding laparoscopic ports, changing the standard position of the ports, changing the patient’s or surgeon’s position, or converting to an open technique.

Incidental findings of true LSG during cholecystectomy should not preclude a laparoscopic approach. It requires meticulous dissection and advanced surgical skills to perform a safe cholecystectomy and avoid inadvertent biliary injury. Although modifications to the laparoscopic technique will help in the safe removal of the gallbladder, a standard laparoscopic approach is still feasible in most cases. Conversion to open surgery may be considered if the biliary anatomy cannot be clearly identified.

## Introduction

The biliary tree consists of intrahepatic bile ducts, extrahepatic bile ducts, and the gallbladder. Anatomically, the gallbladder is located anteriorly on the undersurface of liver segments IV and V [1]. The anatomy of the biliary tree is complex with anatomical variations that can be found in about 50% of the patients [2]. Among these variations, the incidence of left-sided gallbladder (LSG) has been reported to range from 0.2% to 1.1% [3]. It is essential for the surgeon to understand the biliary anatomy and its variations, as it is crucial to perform safe laparoscopic cholecystectomy and avoid iatrogenic biliary injury.

True LSG is the most common type of LSG without situs viscerum inversus (woSVI) [4]. In true LSG, the gallbladder is situated to the left of the round ligament rather than to the right, as in the normal anatomical position. Patients with true LSG who present with symptomatic gallstones exhibit the same clinical picture as those with a normally located gallbladder [3,5], making it difficult to detect this anomaly clinically. This lack of obvious signs may not prompt surgeons to request further imaging. Based on clinical assessment and ultrasonography, which has low sensitivity for detecting true LSG [5], surgeons might proceed with laparoscopic cholecystectomy without anticipating this anomaly.

## Case presentation

A 33-year-old man visited the surgery clinic complaining of episodes of epigastric pain that had occurred four times over the past year. The pain was postprandial (mainly after fatty meals), was severe and constant in nature, affected the patient’s daily activities, lasted for several hours each time, and radiated to the back. It was associated with nausea but no vomiting and was relieved by diclofenac sodium (sachets or injection). The patient had no history of dark urine, pale stool, or pruritus. He also had no history of medical illnesses, allergies, or previous surgeries; was a non-smoker; and had a positive family history of gallstones.

Upon physical examination, the patient showed no pallor or jaundice. He was afebrile with normal vital signs. The abdomen was soft with no peritonism, although there was mild epigastric tenderness.

A complete blood count, kidney function tests, and liver function tests were all normal. Ultrasonography revealed a single large gallbladder stone, normal wall thickness, no pericholecystic fluid, no signs of acute cholecystitis, a normal common bile duct diameter, no intrahepatic dilatation, and a normal liver. The gallbladder position was not visualized on ultrasonography.

**Figure 1:**
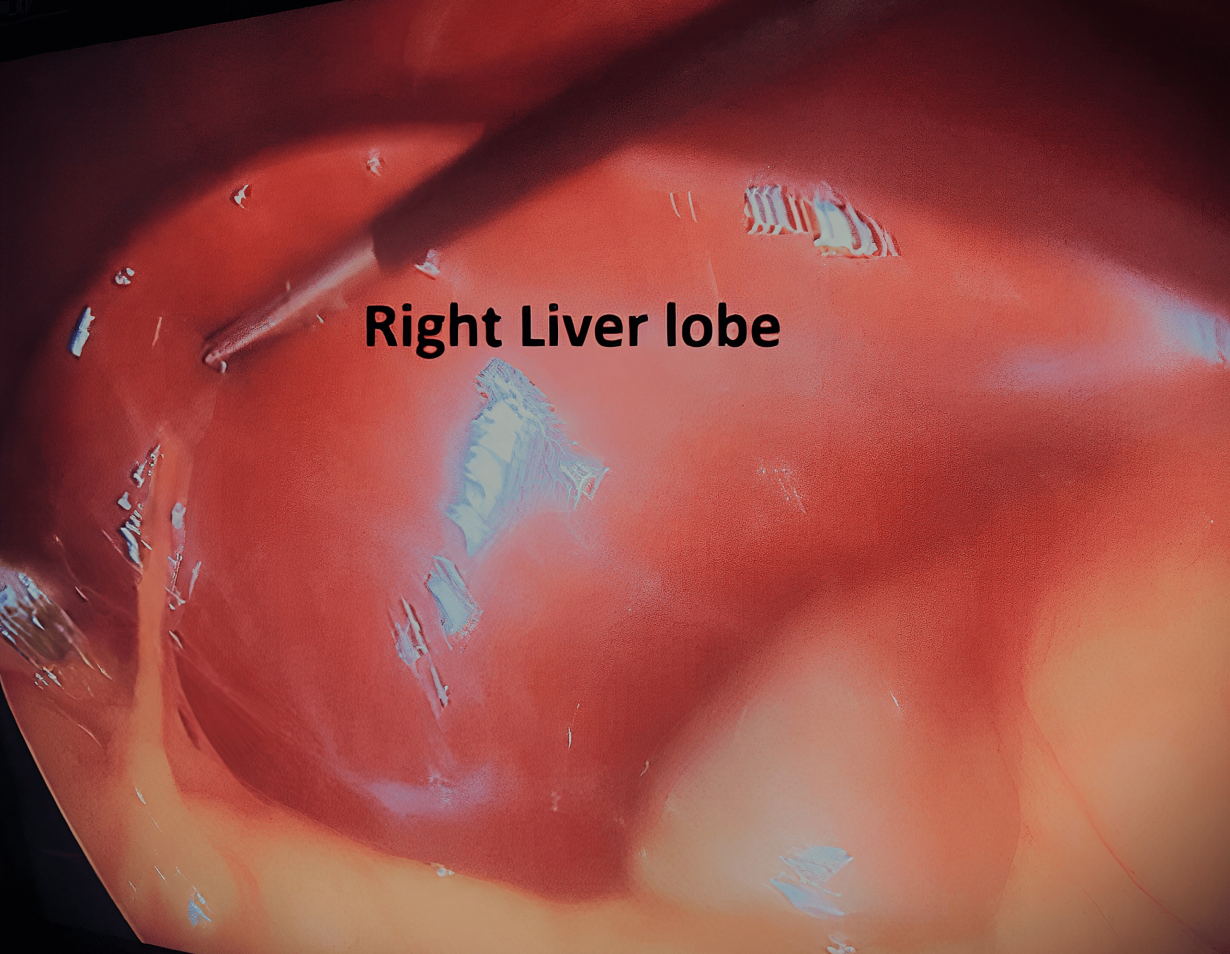
Right liver lobe with no gallbladder in the normal position

An elective laparoscopic cholecystectomy was scheduled, with prophylactic antibiotics administered prior to surgery. Under general anesthesia, the procedure began. A supraumbilical trocar was inserted using an open technique, and pneumoperitoneum was induced. Three additional trocars were placed as follows: a 10-mm subxiphoid trocar, a 5-mm trocar at the tip of the right costal margin in the midclavicular line, and a 5-mm trocar in the right midaxillary line (Figure 1). 

During the exploration of the abdominal cavity, no abnormalities were observed. The gallbladder was not visualized in its usual anatomical position, and an empty right liver lobe was noted (Figure 1).

**Figure 2:**
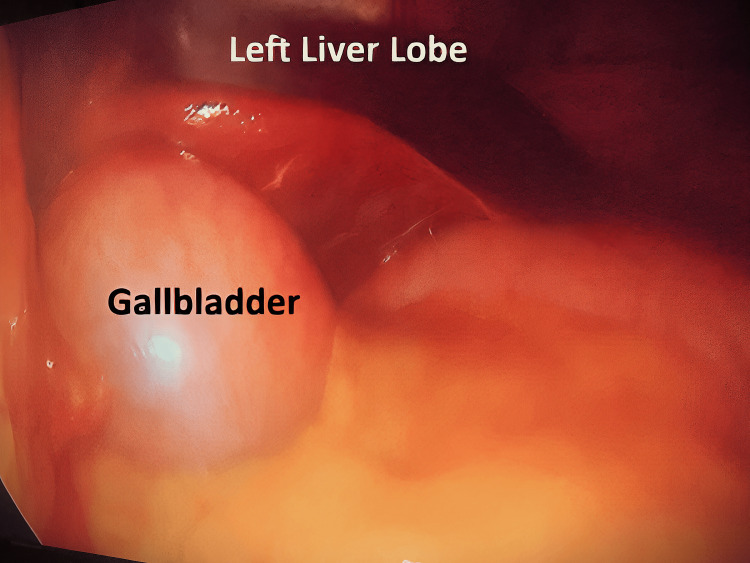
Gallbladder in the left lobe

**Figure 3:**
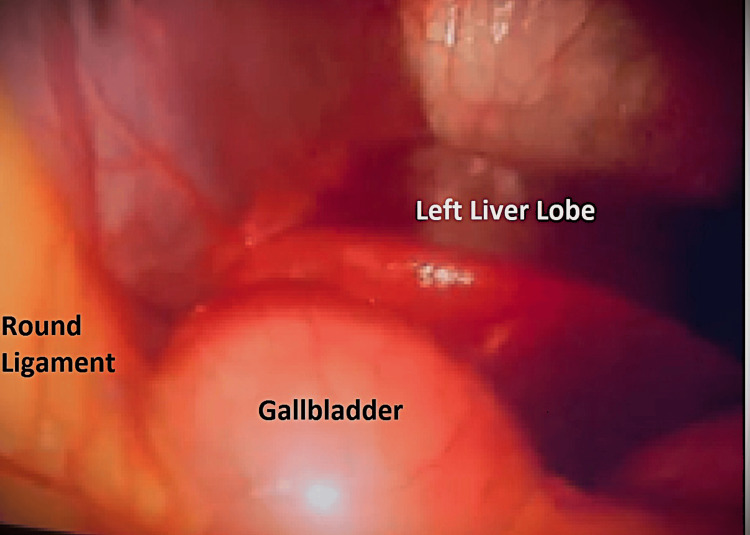
Gallbladder to the left of round ligament

**Figure 4:**
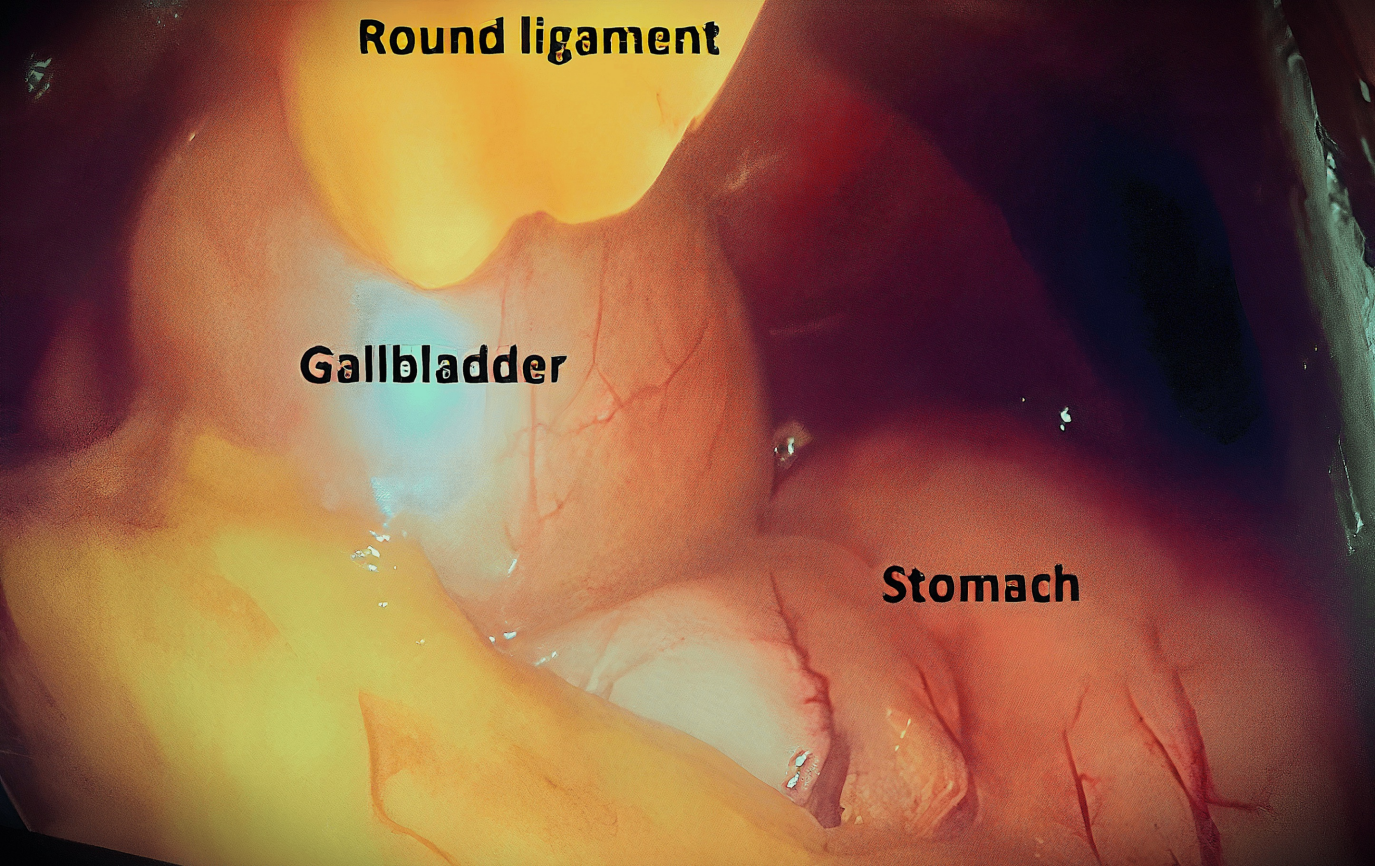
Gallbladder to the left of Round Ligament.

The diagnosis of true LSG was made when the gallbladder was found in the left lobe of the liver (Figure 2), to the left of the falciform and round ligaments (Figures 3, 4). Standard trocars' positions were used as there was no expectation to face this anomaly. No added trocars were inserted.

**Figure 5:**
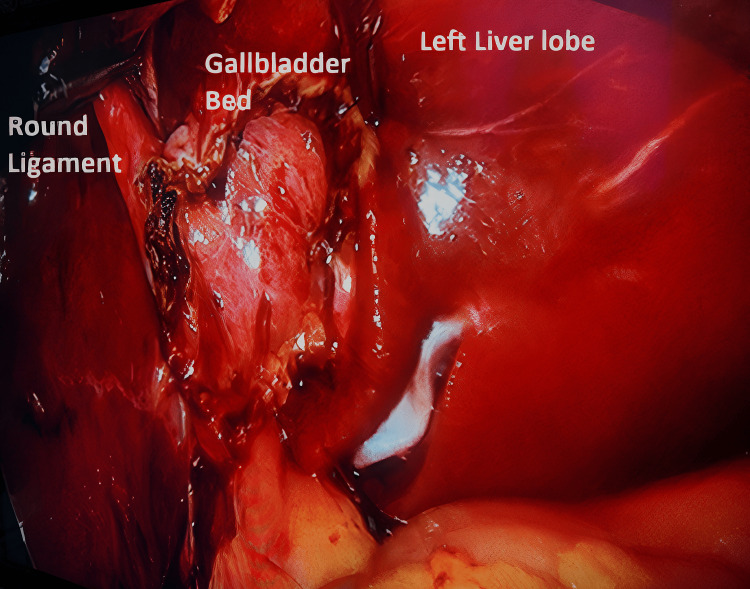
Gallbladder bed to the left of round ligament

An assistant grasper is used to retract the round ligament upward rightward. Excellent access to the Calot's triangle was gained. Through the standard working trocars, delicate dissection of Calot’s triangle was initiated, considering the possibility of associated biliary and vascular variations. A critical view of safety was achieved, so an intraoperative cholangiogram was not done. The cystic duct and cystic artery were identified in normal anatomical locations; then they were clipped and cut. The gallbladder was then dissected from the liver bed (Figure 5) and removed via a retrieval endobag through the epigastric port. The remainder of the operation was carried out as per routine cholecystectomy. The field was dry with adequate hemostasis. A drain was inserted, the abdomen was deflated, and the wounds were closed.

The patient recovered well with a smooth, uneventful postoperative course. He tolerated oral intake and passed bowel motions normally. The drain was removed on the second postoperative day, and the patient was discharged home without complaints. Two weeks later, he was seen in the clinic and reported no complaint. He had normal oral intake, normal bowel motion, and no clinical signs of jaundice or intraabdominal collection. Laboratory tests, including complete blood count and liver function test, were within normal limits. The histopathology report revealed chronic cholecystitis with no suspicious features.

## Discussion

This case report represents the first documented true LSG in Jordan, where a standard laparoscopic approach with no modifications was used safely. It was approved by the local ethics committee (local code 10/2024) of the Royal Medical Services, Amman-Jordan.

Among the wide range of biliary anatomical variations, LSG is a rare variation, with a reported incidence ranging from 0.2% to 1.1% [3]. Fewer than 150 cases have been reported in the literature since the first published case in 1886 [6]. In a retrospective study of published cases from 1996 to 2014, Abongwa et al. reported 55 cases of LSG without situs viscerum inversus [6,7].

**Figure 6:**
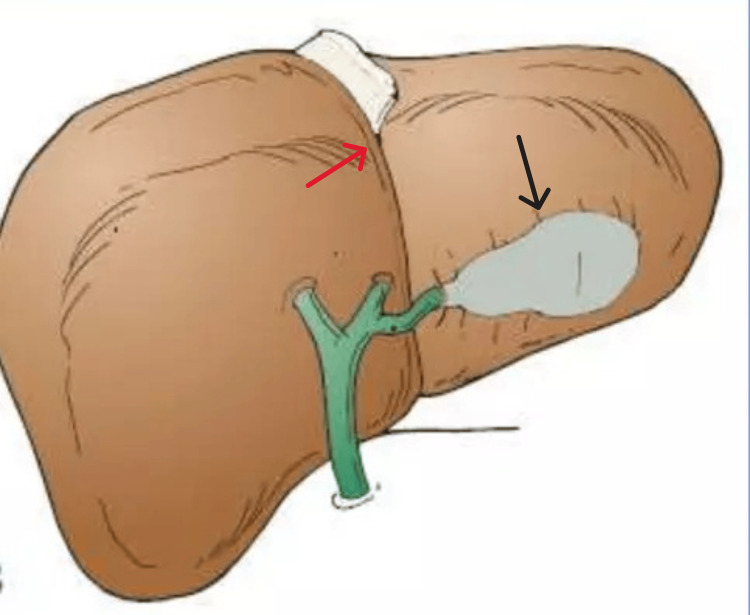
True LSG (red arrow shows the round ligament, black arrow shows the true LSG)

LSG has three variants: LSG with situs inversus, LSG in which the gallbladder is located to the left of an abnormally positioned right-sided round ligament, and true LSG, which is the most common variant and accounts for 83% of cases [3]. In true LSG, the gallbladder is located to the left of the round ligament (Figure 6).

The clinical presentation of symptomatic true LSG is similar to that of patients with a normally located gallbladder, as the vast majority (75%) of these patients present with right upper quadrant pain. This is likely due to the suggestion that visceral nerve fibers do not transpose along with the gallbladder, thereby causing right-sided symptoms [3]. Preoperative imaging studies, such as computed tomography or magnetic resonance imaging, can aid in diagnosing true LSG. However, most cases are discovered intraoperatively because surgeons often rely on ultrasound alone without clinical suspicion of this anomaly. Pereira et al. reported that ultrasound has a positive predictive value of only 2.7% [8].

The importance of discussing this case lies in raising awareness among surgeons about adopting a safe surgical approach and avoiding inadvertent biliary or vascular injury when encountering this anomaly. Discovering true LSG does not preclude a laparoscopic approach if the surgeon can achieve a critical view of safety, either through a standard laparoscopic technique or with modifications, especially if exploration of the abdominal cavity can reveal the presence of true LSG prior to insertion of other trocars. In their review, Nastos et al. [9] reported that true LSG can be managed safely with adjustments to port placement and the use of intraoperative cholangiography.

Modifications to the laparoscopic approach for true LSG may include inserting an additional port, using a fundus-first approach, or employing a mirror-image setup of the trocars. Finally, conversion to an open procedure or referral to a tertiary center where a hepatobiliary surgeon is available should be considered, depending on the surgeon’s experience, especially when there is a high risk of biliary or vascular injury.

## Conclusions

Incidental findings of true LSG during cholecystectomy should not preclude a laparoscopic approach. It requires meticulous dissection and advanced surgical skills to perform a safe cholecystectomy and avoid inadvertent biliary injury. Although modifications to the laparoscopic technique will help in the safe removal of the gallbladder, a standard laparoscopic approach is still feasible in most cases. Conversion to open surgery may be considered if the biliary anatomy cannot be clearly identified.
